# “Why Can’t I Become a Manager?”—A Systematic Review of Gender Stereotypes and Organizational Discrimination

**DOI:** 10.3390/ijerph16101813

**Published:** 2019-05-22

**Authors:** Ana M. Castaño, Yolanda Fontanil, Antonio L. García-Izquierdo

**Affiliations:** Department of Psychology, Universidad de Oviedo, 33003 Oviedo, Spain; castanoperez.ana@gmail.com (A.M.C.); fontanil@uniovi.es (Y.F.)

**Keywords:** gender discrimination, stereotypes, managerial positions, organizational justice, systematic review, decent work, equal employment opportunities, healthy organizations

## Abstract

Women continue to lag behind for accessing managerial positions, partially due to discrimination at work. One of the main roots of such discrimination is gender stereotyping, so we aim to comprehend those biased procedures. First, we have analyzed those highlighted gender lawsuit cases in the scientific literature that have dealt with stereotypes both in the American and the European work contexts. Second, meta-analytic studies regarding organizational consequences of gender stereotypes have been synthetized. Third, gender stereotypes have been grouped by means of a content analysis of the existing literature after processing 61 articles systematically retrieved from WOS, SCOPUS, and PsycINFO databases. As a result, a taxonomy of gender stereotypes has been achieved evidencing that descriptive and prescriptive stereotypes have an impact on decision-making procedures through the apparently perception of women as less suitable for managerial positions. Moreover, we offer a deep explanation of the gender discrimination phenomenon under the umbrella of psychosocial theories, and some measures for successfully overcoming management stereotyping, showing that organizational culture can be improved from both the perspective of equal employment opportunities and the organizational justice frameworks for reaching a balanced and healthier workplace.

## 1. Introduction

A controversial scenario regarding women’s access to the labor market has created a challenge for organizational management. Building sustainable organizations concerned with well-being [1], and combating gender discrimination are at the forefront for promoting decent work worldwide (e.g., [2,3,4]). Decent work is closely related to the concept of healthy organizations, where profits and organizational effectiveness is fostered through employee well-being recognizing diversity as an opportunity (e.g., [5]). In this regard, management should be interested in achieving decent work not only because it is a human right, but also because wasting human talent due to gender discrimination opposes achieving economic progress [2]. As a consequence, management styles in line with social corporate responsibility, sustainability, and organizational ethics are required in order to respect particularities of each employee regardless of gender and, thereupon, in order to raise the employability promotion of women (e.g., [5]). However, despite the aforementioned, an equal environment with no discrimination based on sex (e.g., [6]) has not been efficiently achieved yet, especially for accessing managerial positions (e.g., [7,8]). This inequality has led to work settings characterized by a lack of organizational justice (e.g., [9]), far from the aims of the International Labor Organization’s Decent Work Agenda [10], which in turn has been found to be associated with a lack of well-being, dissatisfaction, and burnout (e.g., [11,12,13]). Consequently, this has led to the maintenance of a lower quality of life and work, especially for females (e.g., [14]).

The fact of the matter is that gender discrimination emerges from a wide variety of barriers, both in the macro-societal (e.g., systematic sexism related with statistical discrimination models based on average performance prejudice which discourages discriminated groups from taking part in market [15]), and the micro-individual levels, which are reproduced into organizational structures (e.g., [16,17]). In order to support organizational trends involved in coping with the gender discrimination phenomenon, we have focused on the comprehension of how management could bias organizational decisions by means of gender stereotypes. Gender stereotypes refer to the historical gender and role division traditionally assigned in the work setting (e.g., [18]), and they could be the basis for both individual biased decisions and for discrimination in the organizations. Stereotypes could be defined as [19] “beliefs about the characteristics, attributes, and behaviors of members of certain groups” (p. 240). These beliefs reflect more generalizations than individual qualities [20], and in consequence their related prejudices may easily and somewhat subtly lead to discriminatory behaviors in the workplace (e.g., [21]), mainly by biasing human resources managerial decisions (e.g., recruitment and selection, promotions, performance assessments), and probably acting as a trigger for the glass ceiling effect, preventing females’ promotion to higher ranking positions [22].

As [17] has suggested, most of the literature dealing with women’s barriers for advancement have not focused on the roles of men, leaders and/or employers, but on the role of women for overcoming hidden and subtle barriers. Hence, the main objective here is to go further in understanding the difficulties experienced by women in (and accessing) managerial positions because of management stereotyping from the complementary social and psychological research approaches [23], in order to prove directions to create bias free and healthy work settings enhancing the employability promotion of women. This objective will be fulfilled through a systematic review of gender stereotypes which help us for drawing robust conclusions, for explaining how and why existing studies contribute to theory and to future research [24], and for summarizing evidence [25]. In this sense, following the guide for systematic reviews by [24] the present paper covers a qualitative narrative review (i.e., a synthesis of the results of quantitative studies with no reference to the statistical significance) and a meta-synthesis (i.e., a synthesis of qualitative studies in order to locate key themes, concepts, and theories) looking for a sound knowledge of gender stereotyping in the labor context on the basis of the following criteria: (i) a higher legal criteria, (ii) a consolidated meta-analytic criteria, and (iii) an empirical criteria by revealing how gender stereotypes have been studied in the scientific literature. Thus, this review begins with an introduction section analyzing those gender lawsuit cases which have been highlighted in the scientific literature that have dealt with stereotyped beliefs both in the American and the European work settings. Then, gender differences have been synthetized by means of the review of published meta-analyses. Later, we carried out a content analysis of gender stereotypes that could hinder women’s presence in managerial positions following the qualitative analysis guidelines by [26]. As a result, a taxonomy which offers a deep explanation of the gender discrimination phenomenon under the umbrella of psychosocial theories has been obtained. Finally, taking the conclusions from all the aforementioned into account, a theoretical ground for overcoming management stereotyping is proposed. This theoretical ground would enhance the advancement of our understanding of how a balanced and healthier workplace can be achieved from both the perspective of equal employment opportunities and organizational justice frameworks.

### 1.1. Lawsuit Cases of Gender Discrimination by Means of Stereotypes in the US and the European Union

The evolution of the presence of women at the workplace has run parallel with the enactment of legal dispositions and Government policies (e.g., [9]), as it is reflected in the legislative development about equality between women and men in the US (e.g., [27]) and in the European context as well (e.g., European Council Directives regarding the principle of equal treatment in employment and the access to and supply of goods and services: [6,28]).

To achieve a healthy work setting built from an equal employment opportunities context beyond legal requirements appeared to be at the forefront of the global social agenda over recent years (e.g., [8]). Consequently, gender discrimination in the workplace is progressively less socially admitted and perceived as a lack of social progress which runs counter the idea of managing diversity for the employability promotion of women, as already discussed in the framework of the decent work (e.g., [3,5,10]). Nonetheless, only 5.2% of chief executive officers in S&P 500 Companies are women [29], even and in the EU-28, female directors on boards was 23.3% in 2016 so the European Commission is urging its member states to increase the presence of women on boards to reach at least the 40% of corporate directors [30]. Likewise, the percentage of women in employment classified under the International Standard Classification of Occupations as managers for OECD and non-OECD countries is below 9% [31]. As a matter of fact, the number of gender discrimination cases, both in courts in the European and North American contexts has continued to be remarkable.

**Figure 1:**
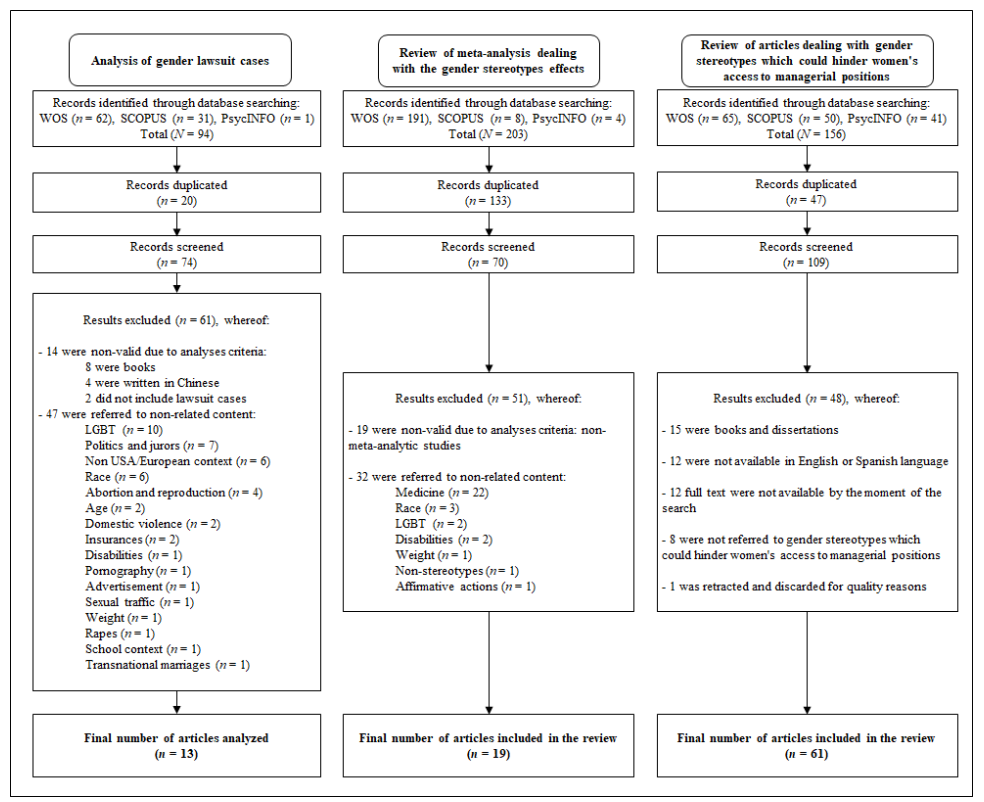
Systematic review flow charts.

With the aim of analyzing to what extent gender stereotypes impact on legal decisions, gender discrimination lawsuit cases highlighted in the scientific literature have been analyzed. First of all, we set up a search strategy for retrieving the articles for being analyzed (e.g., S1 Protocol). The keywords “stereotype”, “gender”, and “Supreme/European court” were used in WOS, SCOPUS, and PsycINFO databases from the beginning of the database records to the date of the search (i.e., 22nd January of 2019). Only US Supreme and European Court decisions have been chosen due to the greater impact of the North American and European Union legislation regarding equal employment opportunities worldwide (e.g., [9]). High Court’s decisions generate jurisprudence and criteria used to interpret cases in both, the Common Law and the Case Law legal systems. We have not considered here inferior court cases, as we were looking in this manuscript for well-established interpretation criteria able to generate strength knowledge. As outlined in Figure 1, retrieved articles (*n* = 94) were explored looking for duplicated articles (*n* = 20) those of which were removed. Then, titles and abstracts were analyzed for the decision to include/exclude from the analysis. If the decision could not be made by the title and the abstract, the full article was retrieved and further screened. Moreover, in order to ensure the inclusion of only objective related results, those referring to lawsuits not dealing with stereotypical gender discrimination at the workplace were not included. More on this, books and results written in a different language from English and Spanish were not included either for guaranteeing both the quality and understanding of the analyzed content. Dealing with non-valid results for analysis, eight were books, four were written in Chinese, and two did not include gender lawsuit cases; and dealing with non-related content, a total of 47 results were not included. These discarded results were referred to topics such as LGBT (*n* = 10), politics and jurors (*n* = 7), non USA/European context (*n* = 6), race (*n* = 6), abortion and reproduction (*n* = 4), age (*n* = 2), domestic violence (*n* = 2), insurances (*n* = 2), disabilities (*n* = 1), pornography (*n* = 1), advertisement (*n* = 1), sexual traffic (*n* = 1), weight (*n* = 1), rapes (*n* = 1), school context (*n* = 1), and transnational marriages (*n* = 1). Summing up, 61 results were excluded, resulting in a total of 13 articles finally included for analysis (e.g., S2 References of articles included in the analysis of gender lawsuit cases).

Data analysis was carried out by means of reading and analyzing the articles looking for organizational European and North American gender lawsuit cases related with gender stereotypes. All the lawsuit cases which fulfilled the aforementioned consideration were written down and then analyzed. It should be noted that only those English abstracts where several European and North American Court cases were cited were considered. After reviewing the references to 249 cases, 51 Supreme Court cases and 18 European Court cases were deeply analyzed. The court decisions and the relevance of the cases were analyzed in terms of stereotyping and the labor context only cases, resulting in a total of 18 cases regarding the Supreme Court of the U.S., and 7 cases regarding the European Court. Table 1 and Table 2 summarize these lawsuit results.

Regarding the American case, some milestones are noteworthy in the context of affirmative actions and gender discrimination. To begin with, the *Price Waterhouse v. Hopkins* case was the first Supreme Court case to use psychological research on sex stereotyping in which a social psychological expert testified [32], being gender discrimination recognized when a woman was refused for being partnership. In a similar way, the *Schlesinger v. Ballard* and the *Johnson v. Transportation Agency* cases were also milestones for striking gender stereotypes in promotion processes. Moreover, there are other relevant cases that have struck with barriers for women at the workplace due to motherhood stereotypes (e.g., *Phillips v. Martin Marietta Corp.*, *Young v. UPS*) and gender segregated work domains (e.g., *Mississippi University for Women v. Hogan*, *UAW v. Johnson Controls, Inc*., *United States v. Burke*, *U.S. v. Virginia*). These cases have set precedents about the rights of women, nevertheless, gender discrimination still had taken place in Supreme Court decisions dealing with promotion (e.g., *Personnel Administrator of Massachusetts v. Feeney*, *Texas Dept. of Community Affairs v. Burdine*, *Wal-Mart v. Dukes*), motherhood (e.g., *Wimberly v. Missouri Labor, Industrial Relations Commission*), and gender segregated domains (e.g., *Bradwell v. Illinois*, *Dothard v. Rawlinson*, *Rostker v. Goldberg*, *Vorchheimer v. School District of Philadelphia*, *Washington v. Gunther*). All in all, 25,605 claims which alleged sex-based discrimination were filed in 2017 [33].

With reference to the European context, it is worth highlighting the *Kalanke v. Bremen* and the *Marschall v. Land Nordrhein-Westfalen* cases as controversial Court decisions where affirmative actions allowed by [28] were not supported by the European Court of Justice. Following [34], positive actions allowed by this Council Directive for increasing the number of women in managerial positions were interpreted from a limited perspective, maintaining gender discrimination and the appearance of a preference in favor of women. Moreover, there are other European Court cases which illustrate the perpetuation of gender stereotypes by considering women as the partner responsible for parental duties (e.g., *Petrovic v. Austria*). Fortunately, in other European cases the Court struck the perpetuation of motherhood gender stereotypes (e.g., *Markin v. Russia*, *Roca Álvarez v. Sesa Start España ETT SA*) and the gender segregation of work domains (e.g., *Emel Boyraz v. Turkey*, *Karlheinz Schmidt v. Germany*) by recognizing gender discrimination due to these traditional differentiations between women and men with no reasonable justification.

In conclusion, half of the 18 Supreme Court cases have broken with gender stereotyped beliefs, whereas the other half remaining cases have been found to be decided on the basis of gender stereotypes. Similar to the North American cases, it has been found in the European Court cases that four out of the seven analyzed cases have broken with stereotyped beliefs, whereas the three remaining cases have not. These findings challenge the adequate consideration of stereotyping in legal decisions, perpetuating traditional gender roles and setting women aside from certain jobs and social status. Even worse, some affirmative actions for compensating women disadvantages are not clear whether struck or perpetuate gender division. This lack of consensus regarding what constitutes gender discrimination on the basis of gender stereotypes from the legal approach call into a serious question the effectivity of equality efforts and the long road ahead to achieve what can be considered a complete and effective healthy and equal employment opportunities context (e.g., [35]). The fact is that legal dispositions on their own seem to be not enough, but how individual decisions are made should be considered for explaining how gender discrimination emerges. In this regard, for a further analysis of gender stereotypes as cognitive activators that could easily impact on women’s presence in managerial positions, the next section deals with their effects on organizational decisions which have been actually found through the scientific literature.

### 1.2. Organizational Effects of Gender Stereotypes: A Meta-Analytic Literature Review

In order to clarify to what extent gender stereotypes could eventually impact on women presence in managerial positions, meta-analyses dealing with gender differences have been synthetized in this section.

Several keywords were combined in WOS, SCOPUS, and PsycINFO databases taking into account the different terminologies that could be used for referring to meta-analytic studies in the academic literature dealing with gender stereotypes effects for women accessing/in managerial positions (e.g., S1 Protocol). Specifically, the terms “meta-analy*”, “gender/female/women”, “stereotype/discrimination”, and “leader/manag*” were combined as keywords resulting in 12 combinations for searching from the beginning of the database records to the date of the search (i.e., 21 January 2019). As shown in Figure 1, retrieved articles (*n* = 203) were explored looking for duplicated articles, which represented a high number of the total due to the similarities of the terms used for the searches (*n* = 133). After removing the duplicates, titles and abstracts were analyzed for the decision to include or not the article for the analysis. If the decision could not be made by the title and the abstract, the full article was retrieved and further screened. Decision for inclusion was taken on the ground of two main criteria: (i) only quantitative meta-analytic results, and (ii) only studies dealing with gender discrimination and stereotyping were considered; that is, those studies dealing with gender discrimination due to other topics different from gender stereotyping and qualitative reviews were refused. As a result, a total of 51 results were excluded from the analysis. Specifically, 19 results were not meta-analytic studies and 32 did not present related content. These discarded results were about topics such as medicine (*n* = 22), race (*n* = 3), LGBT (*n* = 2), disabilities (*n* = 2), weight (*n* = 1), non-stereotypes content (*n* = 1), and affirmative actions (*n* = 1). Finally, a total of 19 articles were included for the review (e.g., S3 References of articles included in the review of meta-analyses). Results of these 19 meta-analyses are summarized in Table 3.

First results are about the absence of differences between women and men. As we can see in Table 3, several studies have pointed out that men and women do not differ neither in cognitive variables [36], nor in terms of being perceived as better due to the physical attractiveness [37], nor in leadership effectiveness and likelihood to initiate negotiations when other variables were considered [38,39]. Specifically, some authors have not found evidence for sex differences in interpersonal, task-oriented, and transactional leadership (e.g., [40,41]), nor in vulnerable narcissism [42]. Even, it has been pointed out that the higher tendency of female leaders for the transformational leadership style could be an advantage for women because transformational leadership has been found to be related to leaders’ effectiveness [43].

Second, we are now to show those results that have found differences between women and men. Despite [44] having found that there was no relationship between sexism and overall workplace discrimination, it seems that effectiveness comparisons favor men [45]. In this sense, [46] found that there is a positive association between having more women on boards of directors and overall financial performance; nonetheless, this association was negative for short-term market returns [47] which brings us the idea of men as being more prone to risk taking, and a negative relationship was found between gender diversity and task performance in countries with low gender egalitarianism [48].

Moreover, we have found in the analyzed studies: (i) an overall masculinity consideration tendency of leader stereotypes [49]; (ii) a higher tendency of men to possess agentic traits whereas women tended to possess higher levels of communal traits [50]; (iii) a higher preference of men for male-dominated jobs [20]; and (iv) a women performance weakening because of a stereotype threat [51]. Taking these results into consideration enables a better understanding of the found evidence for a tendency to evaluate female leaders less favorably than male [52], and the worse hireability and likability of dominant women in comparison with men [53].

Summing up this section, gender stereotypes may influence human resources managerial decisions in a wide variety of ways resulting in female leadership underrepresentation, and contributing to create unbalanced and unhealthy workplaces. In this regard, the present paper focuses below on a qualitative review which specifically aims to unveil the broad variety of gender stereotypes that could hinder women’s presence in managerial positions by means of a content analysis of gender stereotypes in the scientific literature.

## 2. Materials and Methods

A review of the gender stereotypes cited in the literature has been carried out in order to achieve a full view of the broad variety of gender stereotypes. This review has been conducted through the following three steps detailed below.

The first step was a systematic search for articles referred to gender stereotypes (e.g., S1 Protocol). A total of 156 scientific results were retrieved from WOS, SCOPUS, and PsycINFO databases for the period 2009 to 2014, using “woman manager” and “gender stereotypes” as keywords (Figure 1). The initial year for the search was established in 2009 with a view to covering an adequate period of time after the enactment of the main legal gender dispositions regarding equal employment opportunities (e.g., [6,54,55]) as well as the gender mainstreaming approach on gender equality standards and mechanisms reinforced after the 2007 Recommendation of the Ministerial Committee to member states (e.g., [56]) and the post-crisis period. Moreover, references to gender stereotypes prior to 2014 were taken as covered by means of the literature review analysis of the articles. The search scope was broadened with no country limits being set in the databases in order to be as exhaustive as possible in retrieving stereotyped beliefs.

After excluding 47 duplicates, the title and the adequacy of the content of the remaining results were analyzed and a total of 48 results were excluded. Only journal articles dealing with stereotypes which may affect women’s access to managerial positions were included, discarding books and dissertations. In addition, only English and Spanish articles were taken into account for ensuring the reliability and quality of the content analysis. Specifically, 15 results were discarded as they were books and dissertations, 12 results were not available in English or Spanish language, and 8 results were not referred to gender stereotypes which could hinder women’s access to managerial positions. Moreover, 12 results were not available by the moment of the search so they were not included for analysis. Finally, one article was excluded because of quality restrictions as it was retracted by the journal. As a result, 61 journal articles were incorporated into the content analysis (e.g., S4 References of articles included in the content analysis). Table 4 shows the number of included articles by journal. Once the content of the articles (i.e., introduction, results, and discussion sections) were reduced and separated into 1150 units of analysis (i.e., words and pieces of text referring to gender stereotypes which could negatively affect women), a total of 89 themes referring to gender stereotypes which may harm women were identified as a practical way for grouping the units. As no new themes were identified in the retrieved articles for 2014, the theoretical saturation criterion (e.g., [57]) was considered fulfilled for the 2009–2014 time span, and the 12 pending articles unavailable at the moment of the search were neither included. Finally, the analysis of the themes facilitated the identification of the preliminary categories for grouping and coding the units of analysis [26].

As a second step, an inter-rater agreement procedure was carried out for resolving any discrepancies in coding the units of analysis according to the grouping procedure. Those categories and its description were submitted to an inter-rater procedure with two researchers, resulting in a Kappa index (calculated via the SPSS software, version 22) for the agreement of content equal to 0.53. Given the low value of the index of agreement, the groups for coding were re-elaborated taking into account all the comments of the researchers reaching after this process a Kappa index of 0.80. Following a new review, the final Kappa index was 0.86, which according to [58] signifies an almost perfect strength of agreement.

Next, the grouping of the units was improved by including domains, categories, and subcategories. Despite the majority of stereotypic gender beliefs have been usually grouped into communal and agentic attributes [20], the descriptive/prescriptive stereotypes distinction along the lines of [59] was taken into account also, due to their comprehensiveness in integrating the extended information on this topic. In this sense, and on the one hand, communal attributes, more commonly associated with females, are those referred to as the concern for others, as being helpful, kind, nurturing, emotionally expressive, and affectionate; whereas agentic attributes express a tendency to be assertive, controlling, dominant, ambitious, independent, and confident, and these are more strongly associated with males [60].

On the other hand, descriptive stereotypes refer to traits and attributes that are thought to describe men and women; whilst prescriptive stereotypes refer to the set of attributes and characteristics that describe how men and women should actually be. ‘Descriptive stereotype’ refers, for example, to abilities that cannot be included in communal/agentic categories, and the latter can in fact be included in descriptive/prescriptive categories. Thus, we considered the descriptive/prescriptive distinction as more comprehensive for including communal/agentic categories due to practical reasons for grouping. This structure for grouping underwent a new inter-rater agreement procedure with three researchers. The Fleiss’ Kappa index, calculated using the calculator by [61], was 0.72 for the domains, 0.59 for the categories, and 0.58 for the subcategories. In order to improve the inter-rater agreement, the structure was again reviewed in line with the comments of the researchers. Having this done, the new Fleiss’ Kappa index was, respectively, 0.78, 0.62, and 0.62, which following [58] means a substantial strength of agreement. However, the structure was reviewed, taking into account all the comments and discrepancies that emerged in the agreement process, in order to improve the reliability. This new revision was sent to a new researcher who had not participated in the previous steps. The resulting Kappa index stood at 0.95 for the domains, 0.82 for the categories, and 0.76 for the subcategories. After that, following a new revision, the process achieved a total agreement (Kappa index of 1) and a final structure of domains, categories, and subcategories of gender stereotypes was established for coding and grouping the units of analysis.

Finally, as the use of content analysis is a method at the intersection of the qualitative and quantitative traditions wherein word frequency is an indicator of importance [62], at the third step, the final structure of domains, categories, and subcategories was used for counting and coding the 1150 units of analysis retrieved from the 61 analyzed journal articles.

## 3. Results

Results were the following. The descriptive domain includes units of analysis about personal characteristics which results in women being perceived as less suitable candidates for managerial positions than men. These units of analysis were grouped into five categories. The first category comprises stereotypes about those personality traits which resulted in women receiving worse valuations than men, and were grouped into five subcategories on the basis of the Five Factor Model following the personality definitions by [63] (a unit sample is “attributes and qualities that are associated and expected of women such as kindness”). The second category concerns stereotyped beliefs about abilities which women are able (or unable) to perform, and it is grouped into two subcategories: basic cognitive and applied abilities (a unit sample is “women’s thinking and management capabilities are more limited than those of men”). The third category is about the perceived kind of motivation and resilience of women versus men in managerial positions (a unit sample is “their level of aspirations [of women] to these positions is lower than that of men”). The fourth category refers to stereotyped leadership styles beliefs (a unit sample is “female management style, as opposed to the style of their male counterparts, is categorized as ‘weak’”). Finally, the fifth category is about perceived skills and physical appearance (a unit sample is “the sexy woman in top executive posts arouses fewer positive feelings and more negative ones”).

Going to the prescriptive domain, it includes units of analysis dealing with the subjective desirability of actions, behaviors, and roles of women which make them less suitable for managerial positions than men. These units are also grouped into four categories. The first category is related to the adoption of stereotypical gender characteristics resulting into three subcategories depending on the masculine, feminine, and/or androgynous expected characteristics (a unit sample is “higher pressure on women to act in a more ‘caring’ way, so as not to conflict with their expected gender role”). The second category deals with the belief that women are better suited to occupying specific roles, this category being comprised of two subcategories on the basis of family care roles and household chores (a unit sample is “women are unsuitable for certain jobs due to their assumed family orientation”). The third category concerns beliefs about women being better suited for low status positions, and successful women being a mere token in managerial positions (a unit sample is “association between males and superordinate job roles and between women and subordinate job roles”). The fourth category looks at peer rating stereotypes, according to which women are negatively valued by other women indeed (a unit sample is “women reported preferring male management because they feel too much competition with female bosses”).

Table 5 shows the percentages of the units of analysis retrieved from the 61 analyzed journal articles in which the aforementioned stereotypes appeared. From a total of 1150 units of analysis, 751 (65.30%) are specifically referred to as descriptive stereotypes, and 399 (34.69%) to as prescriptive stereotypes. It must be noted that the most frequent descriptive stereotypes are those referring to personality traits (54.33%) and abilities (30.63%), whereas the most frequent prescriptive stereotypes are those relating to the pressures for adopting female/male/androgynous stereotypical leadership characteristics (36.59%) and roles (28.57%).

The counting has shown that descriptive stereotypes are more frequent than prescriptive stereotypes, which following the word frequency count indicator by [62] stereotypes related to women’s personal characteristics are more important in the literature. Specifically, stereotypes involving personality traits and abilities are the most frequent, but motivation, leadership styles, and even the physical appearance of women are also.

## 4. Discussion

One of the issues that emerges from these findings is that it does not seem to matter if women have a specific personal characteristic or not: both the possession and the lack of a given characteristic could lead to a perceived worse performance in comparison to men, making women apparently less suitable. These descriptive stereotypes may even represent a higher cost for organizations because of the perceived inferior performance of women and the association of women with unsuccessful companies as can be viewed from a blaming and set up to fail perspective in line with the Glass Cliff and the Think Crisis-Think Female phenomena (e.g., [64,65]). However, men are perceived in a much more positive way for managerial positions when they have the same characteristics as women.

Moreover, sometimes women are supposed to adopt prescriptive stereotypes, eventually, masculine, feminine, and androgynous roles, in a double bind manner: if they adopt them, then they would be perceived as worse suited for upper echelons organizational levels. Specifically, in line with the backlash effect [66] and the adoption of roles we can state that: if women adopt masculine roles they are perceived as cold and instrumental, whereas if women adopt feminine roles they are perceived as less competent. Moreover, despite the benefits that androgynous roles bring for perceiving women in managerial positions as more favorable (e.g., [67,68]), women still pay a higher price than men for not being androgynous leaders [69]. However, if men adopt feminine, masculine, or androgynous roles, they no suffer from that double bind. Furthermore, in line with occupational segregation models (e.g., [30]), the prescriptive domain also includes those stereotypes which state that women are more suitable for taking care of the family (a lower status position), which entails a ‘sticky floor’ effect because if women spend their time with family and childcare they could not acquire the same experience as men. This is related to the belief that women are seen as tokens and consequently resulting in more severe judgments as compared to men (e.g., [70]). Moreover, [71] has found even, that men quickly leave (‘stopgappers’) female-dominated workplace contexts because of gender pressures, perpetuating workplace gender status segregation.

Finally, it is worth highlighting the result concerning the peer rating category, whereby women are even more negatively valued by other women. For example, [72] have provided empirical evidence that both men and women are prone to assess conflicts among women as more severe than conflicts among men, and in the case of women it might exacerbate the negative consequences of the conflict in recruitment procedures. On the contrary, the ‘homosocial reproduction’ phenomenon [70] consists of men preferring other men, assisting each other with a social support where women are not allowed to participate, for example, using a protective perspective for justifying this exclusion (e.g., women should not assist to a social meeting alone late in the evening). These characteristics may increase the perception of women as being more conflictive for managerial positions by other women. This peer rating stereotypes category is explained by means of the Queen Bee Syndrome [73], according to which, women who are successful in male-dominated environments could be more likely to oppose other female advancement. This phenomenon is better understood nowadays from the System Justification Theory [74], which advocates a general ideological motive that justifies the existing social order by using stereotypes to bolster the status quo [75], granting a sense that the entire system is fair, legitimate, and justifiable (e.g., [76,77]). What is most important now and probably counterintuitive, is that disadvantaged people in terms of status quo may support, defend, and justify existing social systems to reduce ideological dissonance [78]. That is, inequalities and difficulties in social and employment promotions could in fact be maintained by those who stand to lose the most [79] because they try to minimize irritation and resentment towards barriers that lower their own aspirations, as a way of adapting to the social context [75]. In a similar way, [80] has found that although women are generally aware that they earn less than men, women perceive their wages as just as men do, thus, as long as women perceive lower wages as legitimate, inequalities will not be overcome. Therefore, emotional aspects and personal beliefs could play a more important role in discrimination behaviors than expected and should be considered in future gender research.

As a summary, the broad variety of stereotypes which could impact on human resources decisions hindering women’s presence in managerial positions has been highlighted and, consequently, worsen the quality of life of women by enhancing discriminatory and unfair work settings. All things considered, women could experience reduced well-being not only when accessing to managerial positions, but also when they struggle to keep up (e.g., [81]). Hence, organizations committed to embracing decent work should be able to break with the male dominant leadership status quo and the standing justification for management stereotyping. Otherwise, emerging forms of work requiring from diversity for creating value in future work would not be achieved.

Next, in order to reach a better understanding of the gender discrimination phenomenon by moving from descriptive to explanatory accounts in qualitative research [26], the gender stereotypes grouping has been connected with the main general and gender specific psychosocial theories of discrimination depicted in Table 6, developing a comprehensive taxonomy. For this purpose, it has been specifically analyzed whether the psychosocial theories provided a suitable framework for explaining how those gender stereotypes categories and subcategories lead to gender discrimination.

As shown in the taxonomy of Table 7, not all the theories are powerful enough to explain all the gender stereotypes. On the one hand, descriptive stereotypes are better explained by theories in which good and positive characteristics are attributed to the in-group, the highest status groups and men in general. Thus, these categories and subcategories could be explained by the following theories: the Ambivalent Sexism Theory (AST, [82]), the Group-Based Differential Model (GBDM, [83]), the Lack of Fit Model (LFM, [84,85]), the Role Congruity Theory of Prejudice toward Female Leaders (RCT, [60]), the Stereotype Content Model (SCM, [86,87,88]), the Status Characteristics Theory (SCT, [89,90]), the Social Identity Theory (SIT, [91,92]), and the Think Manager-Think Male Theory (TMTM, [93,94]). On the other hand, prescriptive stereotypes are better explained by theories based on the lack of fit model between women and managerial positions, as well as the lack of congruency between stereotypical female and managerial roles. Hence, these categories and subcategories could be explained by the following theories: the AST, the LFM, the RCT, the SCM, the SCT, the TMTM, and the Status Incongruity Hypothesis (SIH, [95]).

Summing up, specific gender discrimination theories are more focused on prescriptive stereotypes, and general discrimination theories are more focused on descriptive ones. Moreover, as previously suggested, it could be better observed that focusing only on one theory may not be powerful enough for explaining the gender discrimination phenomenon, possibly leading to a misunderstanding of how stereotypes are present in organizations.

Following [96], “understanding the underlying dynamics of discrimination is necessary before organizations can take effective action to reduce it” (p. 25). Specifically, for [97], the acknowledgement of the pervasive nature and functions of gender stereotypes can benefit fair judgments when stereotypes are likely to play a role and, according to the Prejudice Habit Model challenges [98], if people are aware of their bias their likelihood of self-regulation is increased and they could break the prejudice habit [99]. In this regard, these results would be helpful for managers to become aware of the gender stereotypes underlying discrimination against women and to avoid managers to discriminate, either intentionally (e.g., for fear of losing power) or because they are not aware of the stereotypes that influence their decisions (e.g., when managers discriminate from a paternalistic perspective protecting women as the backbone of the family).

### 4.1. Can We Overcome Stereotypes?

As we have seen before, decisions could be impacted by means of gender stereotypes. Consequently, this section is focused on proposing several guiding principles on the basis of equality, equity, and fairness from an organizational justice perspective for guiding bias free organizational decision-making and for guiding future research. The idea behind these guiding principles is that enhancing prosocial behaviors on the part of management would spread to the whole organization attracting employees (e.g., [100]) concerned with diversity and sustainability for improving well-being while ensuring economic development and social equity (e.g., [101]) also. For example, the review by [102], pointed out that the perception of ethical leadership increases organizational justice perception, which in turn has been found to be related with well-being (e.g., [103]). Moreover, practices observed in the workplace are relevant for struggle against unethical behavior in organizations (e.g., [104]) such is the case of gender discrimination. In this regard, it has been found that when leaders are moral, they are better suited for enhancing ethical climate decreasing unethical behaviors of employees (e.g., [105,106,107,108]).

In order to allow managers to evaluate to what extent personnel decision procedures are inclusive and free from discrimination, [109] have proposed some practical recommendations which are related to the principles of opportunity to perform, job relatedness, validity, consistency, congruency, communication, treatment, privacy, ethics, and freedom from bias/stereotyping. We can consider these recommendations as soft measures, (e.g., opportunity enhancement programs), as are perceived more favorable than hard measures (e.g., preferential treatment, when even women show more positive attitudes than men [110]), so we can expect to be implemented more easily into organizations. Consequently, we have proposed several specific guiding principles, summarized in Table 8, on the basis of these organizational justice recommendations. These guiding principles are directly related to the gender stereotypes included in the taxonomy for the achievement of healthier and decent working settings improving human resources managerial decisions by means of bias free and fair employability promotion of women.

The first guiding principle is that assessment processes based on an exhaustive and complete job analysis would buffer the influence of descriptive stereotyped beliefs. This analysis permits the incorporation of relevant characteristics in the decision procedure (e.g., [111]), ensuring the quality of the information and reducing the influence of the descriptive gender stereotyped beliefs. Moreover, competencies have been seen as fair and transparent and so are appropriate when profiling jobs (e.g., [112]). In this regard, a joint evaluation in which the performance of male and female candidates is compared has been successful for making unbiased decisions (e.g., [113]).

The second guiding principle establishes that assessment models should integrate the whole personal working characteristics for preventing the influence of stereotyped beliefs. The use of multiple components is recommended for assessing competencies and other characteristics related to the task and duties, as it could assist in preventing some heuristics as the halo effect, avoiding the focus on just one gender-related positive/negative characteristic which influences the final decision.

Third, and closely related to the first guiding principle, the selection system and the instruments must be the same for all the candidates and must be based on scientific evidence and job analysis, ensuring reliability and validity guarantees. That is because high reliable and valid assessment methods are less liable to be influenced by stereotyped beliefs. On the one hand, consistency and validity of the instruments could avoid inadequate assessment of women’s competencies and skills. The better the criterion-orientation established, the less biased the decision taken, because stereotypes are less likely to being activated. For example, there will be less opportunity for the influence of in–out group stereotypes, for the perceived status and warmth stereotypes, and for the think-manager think-male belief. Following [114], the selection methods with higher reliability and operational validity corrected for the criterion’s reliability and indirect restriction in the predictor values are structured behavioral interviews (α = 0.83, validity: 0.63), and knowledge and cognitive tests (α = 0.80 and 0.83, validity: 0.45 and 0.71 respectively); whereas those methods with lower values and which could be more influenced by stereotypes are merit ratings (α = 0.80, validity: 0.18), non-structured interviews (α = 0.50, validity: 0.14), personality tests (agreeableness: α = 0.79, validity: 0.17; conscientiousness: α = 0.80, validity: 0.30), and references (α = 0.60, validity: 0.26). More on this point, [115] have carried out a meta-analysis of applicant reactions dimensions. In relation with the applicant dimensions more related to the influence of gender stereotypes, results have shown that work samples are the methods better scored regarding favorability, face validity, and opportunity to perform; whereas contacts and honesty tests are those with lower scores. Moreover, cognitive tests are the ones with the highest scores for scientific evidence and privacy dimensions; whilst contacts and honesty tests have the lowest scores respectively. Finally, interviews have the highest scores for the perceived employer’s right to obtain information by using this method, whilst contacts present the worst scores.

The fourth guiding principle points out that job postings and assessment methods which avoid gender-type language and personal questions lessen the impact of prescriptive stereotyped beliefs. The questions included in the process must be respectful of the privacy of candidates, for example, no questions about marital status and family care should be included (e.g., [116]) in order to avoid gender stereotypes related to prescriptive family care roles and motivation stereotypes. As has been already pointed out, keeping women as responsible for family care could harm them when being considered for top positions because managerial roles are seen incongruent with family roles representing a paternalistic view of protection, when there is empirical evidence of the benefits for both male and female employees in fostering supportive workplaces for fathers as well [117]. Moreover, avoiding gender-type language could serve for attracting female candidates (e.g., [113]). The use of stereotyped masculine terms concerning traits and roles in job callings may deter potential female candidates from applying due to the traditional association of these terms with male roles, in contrast to the female expected roles of helping and family care. Inclusive language would be helpful in this regard for dissociating prescriptive stereotypes from top positions.

The fifth guiding principle notes that gender balanced hiring committees would entail better opportunities for avoiding the influence of stereotyped beliefs in decision-making. The human resources processes must ensure transparency and efforts must be done to diversify both the members of hiring committees and candidate hiring pools in order to avoid viewing women as tokens, and easily identifying the potential influence of personal interests favoring the main male in-group against the underrepresented female group. Nonetheless, in a sort of affirmative action which is opposed to male homosocial reproduction of men, the use of targeted employee referrals of women in recruitment processes could increase the number of woman applicants (e.g., [113]).

Finally, the sixth guiding principle suggests that training on how to identify and modify stereotyped beliefs would be helpful for the promotion of equality at the workplace. Decision makers must be aware of bias and heuristics which may influence decision making procedures by means of gender stereotypes. As [118] pointed out, heuristics could help in making decisions easily, but they could also lead to errors as the representative, availability, adjustment, and anchoring ones. For example, stereotype-based lower performance expectations of women could affect how decision-makers attend, recall, and interpret the information which confirms a particular stereotype [119]. Nonetheless, [113] noted that internal audits for checking the number of women in lower positions could make a difference in trying to increase their numbers in higher ranking positions and for fostering intergroup contact between lower and higher levels creating a more diverse organizational climate. Moreover, following [120], as stereotypes are flexible cognitive structures it is possible to learn how to identify and modify inadequate heuristics and transform them in an appropriate way. In addition, following [121], if discrimination is considered an unethical conduct it could be possible to carry out interventions against, such as implementing codes of conduct and fair treatment as a moral imperative in organizations contributing towards a decision makers’ awareness about discrimination behaviors that violate human rights. All of this could be complemented with specific executive training in how to identify and confront gender stereotypes and their consequences [113], in line with a target approach (e.g., [122]).

To conclude this section, we have deemed it appropriate to propose some recommendations for good practices in the workplace which aim to offer managers specific actions for developing the aforementioned guiding principles in line with new management styles concerned with diversity and sustainability (e.g., [5]). As shown in Table 9, these good practices have been grouped into five areas for action: (i) recruitment, (ii) personnel selection and assessment, (iii) organizational culture and relationships with stakeholders, (iv) work–family conciliation and professional development, and (v) other areas. Moreover, these recommendations try to offer a holistic approach but also a perspective which focuses on gender categories of stereotypes. Consequently, all recommendations in Table 9 are followed by those categories for which each concrete recommendation could be better suited.

Regarding the recruitment stage, it would be advisable to analyze the percentage of women inside the candidate pool: if the presence of female and male candidates is not balanced, then the human resources department should proactively look to increase the number of women.

The assessment stage is central to avoiding discrimination, so we are going to give to this point a deeper treatment here. We have followed the [123,124] regarding assessment service delivery which provide guidance for developing just organizational procedures (i) before, (ii) during, and (iii) after the assessment. (i) Before the assessment, considerations should be given to what extent assessment methods may be impacted by systematic biases, as well as the reasons for its use. That is, an analysis of bias free assessment should be carried out before the assessment. Moreover, decision-makers should be trained from a bias free and gender perspective. In this sense, the taxonomy developed in this paper proposes a new and comprehensive categorization of gender stereotypes from a discrimination framework which may be especially helpful in making managers and practitioners aware of their own stereotyped beliefs and could be used to influence changes in organizational culture training for the prevention of gender discrimination as suggested throughout the aforementioned guiding principles. That is, the gender stereotypes taxonomy could be helpful for identifying and explaining these gender stereotypes, hence favoring the fight against gender discrimination. For example, the Women in Science & Engineering Leadership Institute of the University of Wisconsin-Madison have developed successful workshops for increasing the number of hired women as faculty members, one of these workshops being focused on enabling participants to recognize the existence of gender bias and to commit themselves to a behavioral change [125]. In this regard, it has been found in meta-analytic studies (e.g., [20]) that decision makers motivated to make careful decisions tended to rely less on stereotyped beliefs. A similar example is the successful creation of a faculty committee designated through a process of peer education which included a review of the research on gender bias [126]. This faculty committee is called Science and Technology Recruiting to Improve Diversity and Excellence, and its purpose is to improve the recruitment and hiring of women at the University of Michigan. Similarly, the gender stereotypes taxonomy developed here could be helpful to use in organizational interventions to promote human resources managerial decisions based only on accurate and valid information, by means of serving as a checklist for identifying and avoiding stereotyped beliefs. In a similar way, decision-makers and managers should be informed about the benefits that gender diverse managerial teams bring to organizations. Finally, enabling not only men, but also women in the establishment of performance criteria would be helpful for avoiding systematic sexism from the beginning of the assessment stage. Later, (ii) during the assessment, the use of perspective taking (i.e., taking others’ perspectives) has been found as a successful strategy for avoiding biases (e.g., [127]), thus, training of decision-makers from this perspective could be useful for avoiding the impact of stereotypes. Moreover, the use of blind CV in which information about the gender of the candidates is not provided could be also used for reducing the impact of gender stereotypes firstly in the assessment. Then, structured behavioral interviews based on competencies together with the writing of minutes of meeting explaining the competencies of the selected candidate seem to be the most appropriate assessment method for avoiding the influence of gender stereotyped beliefs. Furthermore, a balanced presence of women and men in the decision-making tribunal should be helpful for avoiding homosocial reproduction; besides, it has been found that an exposure to women in high-status roles enhances women’s career avoiding the stereotype threat (e.g., [128]), hence viewing women in decision-making positions could enhance performance of women in the assessment process. Finally, (iii) after the assessment, it would be advisable to compare the presence of women and men regarding different departments and levels inside the organization, as well as comparing retributions, results, etc., in terms of women and men to establish a reference number of women in upper positions to achieve in long-term. In this sense, according to [129] it is possible to empirical assess adverse impact, that is, to assess if members of a protected group (i.e., women) are less likely to be hired than members of a favored group (i.e., men). Thereupon, [130] summarized some statistics which could be used to this end: (i) the four-fifths rule or the 80% rule, which compares the selection rate of the protected group with that of the favored group, where a ratio of 0.80 or greater indicated no adverse impact; (ii) the z test, which compares the selection rate differences between the favored and the protected group by calculating the *z* statistic, where a *z* statistic greater than 1.96 in absolute terms meaning that the rate for one group is greater than for the other; (iii) the Fisher’s exact probability test, according to which the frequencies of passing and failing in each group are compared when the sample is small or samples are unbalanced; and (iv), the chi square, which compares differences in proportions for two or more groups. These results could be combined with internal questionnaires to assess which ones are the perceived barriers for the professional advancement of women to guide action.

With regard to the organizational culture and relationships with stakeholders, [128], have highlighted that “Prosecuting a context, rather than an individual, also has the added benefit of organizational examination and alteration of the whole, rather than a dismissal of a deviant few” (p. 227). In this regard, organizational measures such as establishing gender diversity as an added value for the organization inside its good governance code, the creation of an equality label and the requirement of this equality label for establishing relationships with other stakeholders and for the award of public contracts, as well as establishing a gender inclusive communication code, could be good practices in this regard (e.g., [131]).

Dealing with the work–family conciliation and professional development, management should take into account the work–life harmony model [132] which states that harmony occurs when “the resources gained through work/life enrichment […] are successfully aligned with, and serve to, ameliorate, or alleviate the stressors […] arising from work/life conflict” (p. 15), all in all, looking for the well-being of employees. In this regard, organizational decision-makers should keep in mind that both women and men should be equally responsible of childcare, thus both groups should be benefitted from parental leaving. Moreover, meetings both early in the morning and lately in the evening should be avoided to facilitate conciliating work and family life. Non-linear professional career developments which consider motherhood periods and mentoring programs for women in pre-managerial positions could also be considered as well.

Finally, other areas in which good practices could be implemented are collective bargaining, verifying that both retributions of women and men are equal in the interest of sustainability, profitability, and equality (e.g., [133]). The [134] regarding social responsibility, equality between women and men is considered as relevant for social responsibility and some recommendations are the following ones: including both women and men in upper positions, same retribution for same work, and a gender perspective in the study of work health. Moreover, an advisory service for women inside the organizations which helps them dealing with career development and gender discrimination issues, and which sets female managerial referents breaking with the predominant stereotypical representation of male (e.g., [135,136,137]) could be implemented in line with worldwide social concerns such as the fifth Sustainable Development Goal by the United Nations regarding the achievement of gender equality and empowering all women and girls.

### 4.2. Limitations and Future Research Agenda

As the main caveats of this paper, on the one hand, the time span for the search of articles included in the content analysis is restricted to five years. Nonetheless, as it has been pointed out, the content analysis has been based on theoretical saturation, thus, the number of articles retrieved and the period of time should have been enough for obtaining a relevant and wide ranging sample of gender stereotypes. In a similar way, only those cases cited in the literature have been included for the analysis of gender lawsuit cases, thus there might be a publication bias, but we have focused on those outstanding cases.

On the other hand, we can outline that only the research perspective has been taken into account. Moreover, gender stereotypes have only been analyzed from the point of view of the decision-maker but this is the bottom line. As it has been already noted, stereotyped beliefs could be kept alive even by women trying to minimize irritation towards barriers (e.g., [74]). This together with the stereotype threat, that is, the risk of being judged in light of stereotypes which can undermine women’s performance (e.g., [138,139]), highlights that future research should also be aware of those personal beliefs and emotional aspects which could play an important role for maintaining gender discrimination in the workplace. Additionally, future studies on the development of gender stereotypes measures on the basis of the grouped units of analysis could be investigated.

Finally, could be suggested to take into account the aforementioned practical recommendations for guiding the development of quality and fair human resources procedures, and to test their efficiency for increasing the number of women in top organizational positions.

## 5. Conclusions

In this paper, we sought to shed light on how management could bias organizational decisions by means of stereotypes as the hidden origin of gender discrimination in managerial positions. Firstly, the analysis of lawsuit cases highlighted in the scientific literature has pointed out the controversial interpretation of gender stereotypes as potential causes of the gender discrimination phenomenon at the workplace. Secondly, detrimental effects of gender stereotypes have been corroborated through a review of meta-analytic studies, finding that even perceived advantageous differences could support the likelihood of women being mistreated and not considered for managerial positions. Thirdly, this paper has also shed light on the broad range of the gender stereotypes that subtly influence organizational decisions leading to discriminatory behaviors towards women in managerial positions (e.g., S5 PRISMA checklist is available as Supplementary Materials for more information on the systematic review carried out in this paper). Specifically, it has been evidenced that descriptive and prescriptive stereotypes impact decision-making processes, mainly through the apparent perception of women as being less suitable for managerial positions. As a consequence, workplaces characterized by a lack of justice have been sustained over time, then, it is not surprising that women continue to be overrepresented in low-skilled occupations with negative consequences for health and well-being although they are equally or even more qualified than men (e.g., [3]). Finally, as a result of the integration of the aforementioned stereotypes and the main psychosocial theories of gender discrimination it has been concluded that focusing on a sole theory is insufficient when explaining the gender discrimination phenomenon. That is, the gender discrimination phenomenon can be better understood by means of explaining the full range of gender stereotypes from a comprehensive perspective. In this regard, following the suggested guiding principles and the recommendations for good practices from equal employment opportunities and organizational justice frameworks will presumably contribute to avoiding erroneous decisions by means of providing management with resources and competencies for implementing new styles of management concerned with decent work and the equitable and fair employability promotion of women. Furthermore, it could effectively help in overcoming the gap between equality policies and the organizational context, contributing to answering the question of why women cannot become managers and creating a balanced and healthier workplace.

## Figures and Tables

**Table 1:** Summary of North American lawsuit gender cases.

Case	No.	Description	Court Decision
**Promotion**			
*Price Waterhouse v. Hopkins 1989*	6	Ms. Hopkins was refused for partnership despite her good economical results, arguing she was aggressive.	The court considered that refusing the partnership of Ms. Hopkins constituted gender discrimination. (+)
*Schlesinger v. Ballard 1975*	2	A man complained against a navy’s promotion policy that allows longer time in rank for female officers.	The court held that the different treatment results from the different opportunities for professional service. (+)
*Personnel Administrator of Massachusetts v. Feeney 1979*	2	A woman was ranked below male veterans who had lower scores due to the preference to honorably discharged veterans.	The court found no discriminatory purpose. (−)
*Texas Dept. of Community Affairs v. Burdine 1981*	2	A woman applied for a supervisor position, but a male employee from another division was hired.	The court held that the decision was not based on unlawful criteria and the company was not required to hire female applicants equally qualified. (−)
*Wal-Mart v. Dukes 2011*	2	Several women filed a class action lawsuit for experiencing sex discrimination in pay and promotion.	The court viewed the claims as lacking the necessary commonality to support class certification. (−)
*Johnson v. Transportation Agency 1987*	1	Mr. Johnson was passed over for a promotion in favor of Ms. Joyce on the basis of an affirmative action plan.	The court considered that the Transportation Agency appropriately took into account Joyce’s sex as determinant for the promotion. (+)
**Motherhood**			
*Young v. UPS 2015*	2	A woman was forced to take an unpaid leave, as the company policy required being able to lift up more pounds than the pregnant complainant was advised to.	The court established the criteria unconstitutional. (+)
*Phillips v. Martin Marietta Corp. 1971*	1	A complaint against an employer who refused to hire women, but not men, with young children.	The court held that an employer could not refuse to hire females with young children. (+)
*Wimberly v. Missouri Labor and Industrial Relations Commission 1987*	1	A woman complained for not being rehired after taking pregnancy leave, alleging she had left work voluntarily and without good cause.	The court declared that the denial of her unemployment compensation was not based upon her pregnancy and therefore was not illegal. (−)
**Gender segregated domains**			
*Mississippi University for Women v. Hogan 1982*	4	A qualified applicant was denied the admission to the Mississippi University for Women School of Nursing on the basis of sex.	The court found gender discrimination. (+)
*Bradwell v. Illinois 1873*	2	Lawyer Mira Bradwell was refused a license to practice law because she was a woman.	The court held that women did not have the right to practice law. (−)
*Dothard v. Rawlinson 1977*	2	A complaint against excluding women from positions as prison guards in maximum security facility.	The court held that having women as prison guards would create security and safety problems. (−)
*U.S. v. Virginia 1996*	2	A complaint against the exclusion of women from a military college.	The court held that male-only admissions policy was unconstitutional. (+)
*Vorchheimer v. School District of Philadelphia 1977*	1	A complaint against a public high school restricted to males.	The court held the gender classification. (−)
*Rostker v. Goldberg 1981*	1	A complaint against male only military registration.	The court held the only male military registration. (−)
*Washington v. Gunther 1981*	1	Female county prison guards complained for being paid less than male guards.	The court held that the wage differential is based on a differential based on any other factor than sex. (−)
*UAW v. Johnson Controls, Inc. 1991*	1	A complaint against the prohibition of women, except for infertile women, from engaging in tasks with lead exposure.	The court held that excluding women was sex discrimination. (+)
*United States v. Burke 1992*	1	A case about increasing the salaries of employees only in male dominated pay schedules.	The court held that discrimination constituted a tort-like injury to respondents. (+)

Note: Information of the cases has been supplemented by consulting the Oyez multimedia judicial archive of the Supreme Court of the United States website (https://www.oyez.org/); “+” means “perpetuating gender stereotypes struck”; “−” means “perpetuating gender stereotypes upheld”; No. refers to the number of articles which cite the lawsuit case.

**Table 2:** Summary of European lawsuit gender cases.

Case	No.	Description	Court Decision
**Promotion**			
*Kalanke v. Bremen 1995*	2	Mr. Kalanke was initially proposed for a promotion, however, Ms. Glissman (holding equal qualifications for the job) was finally promoted.	The sentence considered unlawful a positive discrimination action favoring the promotion of a woman. (−)
*Marschall v. Land Nordrhein-Westfalen 1997*	1	A female candidate with the same merits as Mr. Marschall was proposed for a promotion instead of him because of an affirmative action.	The sentence supported the decision of the Kalanke’s case, although the legality of positive discrimination was recognized. (−)
**Motherhood**			
*Petrovic v. Austria 1998*	4	A father was denied parental leave on the basis that only mothers were allowed.	The court denied the parental leave of the father. (−)
*Markin v. Russia 2012*	4	Russian serviceman who was responsible for his children claimed equal parenting rights as women.	The court held that maternity leave to mothers not fathers perpetuates gender stereotypes hindering women’s careers and men’s family life. (+)
*Roca Álvarez v. Sesa Start España ETT SA 2010*	1	Roca Álvarez requested the right to take the parental leave, but his request was refused because the mother of his child was self-employed.	Refusing the father’s leave when the mother was self-employed perpetuated the role of women as the main responsible for parental duties. (+)
**Gender segregated domains**			
*Karlheinz Schmidt v. Germany 1994*	1	A case about fire brigade duty that was compulsory for men only, arguing the protection of women.	The court found a violation of the Convention. (+)
*Emel Boyraz v. Turkey 2014*	1	A woman was denied a position as a security officer in the state-run electricity company because she was a woman.	The court stated that work night shifts and physical force requirements could not in itself justify the difference in treatment between men and women. (+)

Note: Information of the cases has been supplemented by consulting the Judgments of the Court in the InfoCuria for case-law of the Court of Justice website (http://curia.europa.eu/juris/recherche.jsf?language=en), the HUDOC for case-law of the European Court of Human Rights database (https://www.echr.coe.int/Pages/home.aspx?p=caselaw/HUDOC&c=), and the briefs of the cases in https://www.womenslinkworldwide.org/; “+” means “perpetuating gender stereotypes struck”; “−” means “perpetuating gender stereotypes upheld”; No. refers to the number of articles which cite the lawsuit case.

**Table 3:** Summary of meta-analyses regarding differences between women and men.

Reference	Results
**Leadership styles**	
Eagly AH, Karau SJ, Johnson BT 1992	Women were more democratic and less autocratic than men, however there were no differences for either interpersonal style or interpersonal versus task style.
Eagly AH, Johannesen-Schmidt MC, van Engen ML 2003	Female leaders were more transformational than male leaders and also engaged in more contingent reward; whereas men manifest greater transactional active and passive leadership and laissez-faire approaches.
Van Engen ML, Willemsen TM 2004	Men tended to use the traditionally masculine styles and women the traditionally feminine styles: women tended to use the stereotypical feminine styles democratic-versus-autocratic and transformational leadership styles. However, no evidence was found for sex differences in interpersonal, task-oriented, and transactional leadership.
**Leadership idea**	
Eagly AH, Makhijani MG, Klonsky BG 1992	There was a small overall tendency to evaluate female leaders less favorably than male leaders, especially when female leadership was carried out in stereotypically masculine styles, in male-dominated roles, and when the evaluators were men.
Koenig AM, Eagly AH, Mitchell AA, Ristikari T 2011	Masculinity of leader stereotypes was found: (a) male-leader similarity; (b) greater agency than communion; and (c) greater masculinity.
Koch AJ, D’Mello SD, Sackett PR 2015 *	Men were preferred for male-dominated jobs, and male raters exhibited greater gender-role congruity bias than did female raters for male-dominated jobs.
Badura KL, Grijalva E, Newman DA, Yan TT, Jeon G 2018	Men tended to emerge in leadership roles more often than did women. Moreover, men tended to possess higher levels of agentic traits, whereas women tended to possess higher levels of communal traits.
**Effectiveness and performance**	
Eagly AH, Karau SJ, Makhijani MG 1995	Male and female leaders were equally effective. However, effectiveness comparisons favored men for first-level leadership; and female leaders fared better in feminine expected roles such as education, whereas male leader fared better in masculine expected roles such as military.
Paustian-Underdahl SC, Walker LS, Woehr DJ 2014	Men and women did not differ in perceived leadership effectiveness when all leadership contexts are considered.
Schneid M, Isidor R, Li C, Kabst R 2015 *	A negative relationship was found between gender diversity and contextual performance, although no relationship was found with task performance. However, gender diversity has a significant negative relationship with task performance in countries with low gender egalitarianism.
Jeong SH, Harrison DA 2017	Female presence in CEO positions was positively related to long-term financial performance and negatively related to short-term market returns; whereas female presence in top management teams was positively related to long-term financial performance but not to short term market returns.
Hoobler JM, Masterson CR, Nkomo SM, Michel EJ 2018	There was a positive association between having more women on boards of directors and overall financial performance.
**Personality and individual characteristics**	
Hyde JS 2005 *	There were small or non-differences regarding cognitive variables, verbal or nonverbal communication, social or personality variables, wellbeing, motor behaviors, and moral reasoning.
Grijalva E et al., 2015 *	Men tended to be more narcissistic than women, however, men and women did not differ on vulnerable (low self-esteem, neuroticism, and introversion) narcissism.
Williams MJ, Tiedens LZ 2016	Dominance expressed explicitly affected women’s likability, whereas implicit forms of dominance did not. Furthermore, dominant women were found to have worse outcomes on dimensions such hireability. Nonetheless, non significant differences were found regarding men’s and women’s perceived competence.
Kugler KG, Reif JAM, Kaschner T, Brodbeck FC 2018 *	Women were less likely to initiate negotiations than men. However, gender differences were smaller for low situational ambiguity and situational cues, consistent with the female gender role.
**Other criteria: physical appearance, stereotype threat, and sexism**	
Hosoda M, Stone-Romero EF, Coats G 2003 *	Attractive people were found to be better than unattractive on job-related outcomes, obtaining similar values for women and men.
Nguyen H-HD, Ryan AM 2008 *	The overall performance of women stereotyped test takers might suffer from a situational stereotype threat.
Jones KP et al., 2017 *	There was no relationship between sexism and overall workplace discrimination.

* Studies marked with an asterisk are not specifically referred to managerial positions but they have been included as they could hinder women accessing/in managerial positions.

**Table 4:** Journal and number of articles in the content analysis.

Journal	Number of Articles Retrieved Per Journal	Total of Articles Retrieved
*Gender in Management: An International Journal*	7	7
*Gender, Work & Organization*	4	4
*Journal of Managerial Psychology* *Psychology of Women Quarterly*	3	6
*Higher Education* *Human Relations* *Journal of Applied Psychology* *Journal of Business and Psychology* *SAGE Open*	2	10
*African Journal of Business Management* *Applied Psychology* *Australian Journal of Public Administration* *British Journal of Management* *British Journal of Social Psychology* *Estudios de Psicología* *European Journal of Work and Organizational Psychology* *Human Resource Development International* *Human resources for health* *International Journal of Conflict Management* *International Journal of Health Policy and Management* *Journal of Nursing Management* *International Journal of Emerging Markets* *International Journal of Gender and Entrepreneurship* *International Journal of Hospitality Management* *Journal of Applied Social Psychology* *Journal of Business Ethics* *Journal of Business Research* *Journal of International Business Studies* *Journal of Management* *Journal of Small Business and Enterprise Development* *Negotiation and Conflict Management Research* *Organizational Dynamics* *Organization Science* *Psychological Bulletin* *Psykhe* *Revista Psicologia Organizações e Trabalho* *Scandinavian Journal of Management* *Social Forces* *Strategic Management Journal* *Swiss Journal of Psychology* *The History of the Family* *The Leadership Quarterly* *The Spanish Journal of Psychology*	1	34
	Total	61

**Table 5:** Descriptive and prescriptive stereotypes categories counting.

Domains, Categories, and Subcategories of Gender Stereotypes	Units of Analysis Retrieved from the Analyzed Journal Articles	
	Number	%
Descriptive stereotypes		
1. Personality traits	408	54.33
1.1. Agreeableness	137	33.58
1.2. Extraversion	72	17.65
1.3. Conscientiousness	68	16.67
1.4. Neuroticism	50	12.25
1.5. Openness	24	5.88
No subcategory *	57	13.97
2. Abilities	230	30.63
2.1. Applied	205	89.13
2.2. Basic	12	5.22
No subcategory *	13	5.65
3. Leadership styles	79	10.52
4. Motivation	21	2.79
5. Physical appearance	13	1.73
Total	751	65.30
Prescriptive stereotypes		
1. Adopting stereotypical gender characteristics	146	36.59
1.1. Masculine	65	44.52
1.2. Feminine	51	34.93
1.3. Androgynous	13	8.90
No subcategory *	17	11.64
2. Roles	114	28.57
2.1. Family care	59	51.75
2.2. Working home	30	26.32
No subcategory *	25	21.93
3. Status	103	25.81
4. Peer rating	36	9.02
Total	399	34.69

Note: Percentages of categories do not add to 100% because they are referred to their upper domain (i.e., descriptive/prescriptive), and percentages of subcategories are referred to their immediately upper category; * No subcategory is used for units of analysis referred to the general category, as it is not possible to classify them into a subcategory.

**Table 6:** Classification of discrimination theories.

Theory	Discrimination Criterion
General discrimination theories	
-Social Identity Theory -Group-Based Differential Model	A. The categorization of people into in/out-groups
-Status Characteristics Theory -Stereotype Content Model	B. The categorization of people regarding the perceived status and/or warmth
Gender discrimination theories	
-Lack of Fit Model -Think Manager-Think Male Theory -Role Congruity Theory of Prejudice Toward Female -Status Incongruity Hypothesis	C. A perceived lack of fit between the women’s attributes and roles and the managerial requirements
-Ambivalent Sexism Theory	D. Positive and negative feelings about women

**Table 7:** Taxonomy of gender stereotypes and psychosocial theories of gender discrimination.

Gender Stereotype	Psychosocial Theories of Gender Discrimination
Descriptive stereotypes	
1. Personality traits (i.e., agreeableness, extraversion, conscientiousness, neuroticism, openness)	GBDM, LFM, RCT, SCM, SIT, TMTM
2. Abilities (i.e., applied, basic)	GBDM, LFM, SCM, SCT, SIT, TMTM
3. Leadership styles	GBDM, LFM, SCM, SCT, SIT, TMTM
4. Physical appearance	AST, GBDM, LFM, SIT, TMTM
5. Motivation	GBDM, LFM, SIT, TMTM
Prescriptive stereotypes	
1. Adopting stereotypical gender characteristics (i.e., masculine, feminine, androgynous)	LFM, RCT, SCM, SIH, TMTM
2. Roles (i.e., family care, working home)	AST, LFM, RCT, SCM, SCT, SIH, TMTM
3. Status	LFM, SCM, SCT, SIH, TMTM
4. Peer rating	LFM, SCM, TMTM

Note: Ambivalent Sexism Theory (AST); Group-Based Differential Model (GBDM); Lack of Fit Model (LFM); Role Congruity Theory of Prejudice Toward Female (RCT); Stereotype Content Model (SCM); Status Characteristics Theory (SCT); Status Incongruity Hypothesis (SIH); Social Identity Theory (SIT); Think Manager-Think Male Theory (TMTM).

**Table 8:** Summary of guiding principles for the improvement of human resources managerial decisions regarding stereotypes.

Guiding Principles
Guiding principle 1: Assessment processes based on job analysis would buffer the influence of descriptive stereotyped beliefs.
Guiding principle 2: Assessment models should integrate the whole personal working characteristics for preventing the influence of stereotyped beliefs.
Guiding principle 3: High reliable and valid assessment methods are less liable to be influenced by stereotyped beliefs.
Guiding principle 4: Job postings and assessment methods which avoid gender-type language and personal questions lessen the impact of prescriptive stereotyped beliefs.
Guiding principle 5: Gender balanced hiring committees would entail better opportunities for avoid the influence of stereotyped beliefs in decision-making.
Guiding principle 6: Training on how to identify and modify stereotyped beliefs would be helpful for the promotion of equality in the workplace.

**Table 9:** Recommendations for good practices at the workplace for buffering gender stereotypes.

Area	Good Practices	Stereotypes
Recruitment	-Analyze the percentage of women inside the candidate pool looking for balancing the number of women and men.	MO, RO, ST
Personnel selection and assessment	Before the assessment:	
	-Provide evidence of bias free assessment and criterion oriented methods before their use.	PE, AB, PH
	-Carry out workshops for decision-makers in order to identify gender stereotypes.	ALL
	-Inform decision-makers about the benefits of gender diverse managerial teams.	ALL
	During the assessment:	
	-Make decisions from the perspective taking approach.	MO, PR
	-Use blind CV with no information about the gender of the candidates.	ALL
	-Carry out structured behavioral interviews and apply valid and reliable instruments and methods based on competencies.	PE, AB, LE, MO
	-Provide the explanation of the competencies of the selected candidate.	PE, AB, LE, PH, MO
	-Balance the presence of women and men in the decision-making tribunal.	AD, RO, ST, PR
	After the assessment:	
	-Compare the presence of women and men regarding departments and levels.	RO, ST
	-Compare retributions of women and men.	PE, AB, ST
	-Establish a reference number of women in higher positions to achieve in long-term.	MO, RO, ST
	-Empirical assessment of adverse impact.	PE, AB, PH, AD, RO, ST
	-Assess perceived barriers for the professional advancement of women.	ALL
Organizational culture and relationships with stakeholders	-Establish gender diversity as an added value inside the good governance code. -Create and promote an equality label. -Require the equality label for establishing relationships with stakeholders and for the award of public contracts. -Establish a gender inclusive communication code.	ALL
Work–family conciliation and professional development	-Establish equal parental leave for women and men.	AD, RO
	-Avoid meetings both early in the morning and late in the evening.	RO
	-Implement non-lineal professional career development.	MO, RO
	-Establish mentoring programs for women in pre-managerial positions.	LE, MO, ST
Other areas	-Verify that both retributions of women and men are equal.	AB, ST, PR
	-Implement an advisory service for women inside the organizations.	MO, RO, PR

Note: personality traits (PE), abilities (AB), leadership styles (LE), physical appearance (PH), motivation (MO), adopting stereotypical gender characteristics (AD), roles (RO), status (ST), peer rating (PR). When a recommendation is followed by ALL it means that the recommendation is suited for avoiding gender stereotypes categories widely, as the recommendations deals with broad scope topics (e.g., gender, equality, gender diversity).

## Supplementary Materials

The following are available online at https://www.mdpi.com/1660-4601/16/10/1813/s1, S1: Protocol, S2: References of articles included in the analysis of gender lawsuit cases, S3: References of articles included in the review of meta-analyses, S4: References of articles included in the content analysis, S5: PRISMA checklist.
