# Supplementary material for: “Why Can’t I Become a Manager?”—A Systematic Review of Gender Stereotypes and Organizational Discrimination
Source: Int J Environ Res Public Health. 2019 May 22;16(10):1813. doi: 10.3390/ijerph16101813 (PMC6572654; doi:10.3390/ijerph16101813)
Supplement: Supplementary file 1 [file ijerph-16-01813-s001.zip › S2_References of articles included in the analysis of gender lawsuit cases.docx]

**S2_References of articles included in the analysis of gender lawsuit cases**

1. Bornstein S. The Law of Gender Stereotyping and the Work-Family Conflicts of Men. Hastings Law J. **2012**, 63, 1297-344.

2. Bornstein S. Reckless Discrimination. Calif Law Rev. **2017**, 105, 1055-110. doi: 10.15779/Z388P5V86M

3. Brglez M, Novak S, Tkalec S. Stereotyping and Human Rights Law: an (Un)conventional Approach of the European Court of Human Rights. Teorija in Praksa. **2016**, 53(5), 1124-37.

4. Chamallas M. Deepening the Legal Understanding of Bias: On Devaluation and Biased Prototypes. S Cal L Rev. **2001**, 74(3), 747-806.

5. Fiske ST, Bersoff DN, Borgida E, Deaux K, Heilman ME. What constitutes a scientific review? A majority retort to Barrett and Morris. Law Hum. Behav. **1993**, 17(2), 217-33. doi: 10.1007/BF01045940

6. Fredman S. Emerging from the Shadows: Substantive Equality and Article 14 of the European Convention on Human Rights. Human Rights Law Review. **2016**, 16(2), 273-301. doi: 10.1093/hrlr/ngw001

7. Kong YJ. A Study on Affirmative Action and Positive Action as Countermeasures to Employment Discrimination in the U.S. and the E.U. The Journal of Labor Law. **2011**, 21, 229-62.

8. Mezey SG. When Should Differences Make a Difference. Women & Politics. **1990**, 10(2), 105-20. doi: 10.1300/J014v10n02_08

9. Nolasco CARI, Vaughn MS. Judicial scrutiny of gender-based employment practices in the criminal justice system. J Crim Justice. **2011**, 39(2), 106-19. doi: 10.1016/j.jcrimjus.2010.11.002

10. Sung JT. The Latest US Federal Supreme Court Attitude of Discrimination of Pregnant Women in the Workplace and the Implications of our Legal System. Study on The American Constitution. **2016**, 27(2), 157-92.

11. Timmer A. Toward an Anti-Stereotyping Approach for the European Court of Human Rights. Human Rights Law Review. **2011**, 11(4), 707-38. doi: 10.1093/hrlr/ngr036

12. Timmer A. Judging Stereotypes: What the European Court of Human Rights Can Borrow from American and Canadian Equal Protection Law. Am J Comp Law. **2015**, 63(1), 239-84. doi: 10.5131/AJCL.2015.0007

13. Yuracko KA. Soul of a Woman: The Sex Stereotyping Prohibition at Work. Faculty Working Papers. **2012**, 207, 1-65.

**Lawsuit gender cases:**

Bradwell v. Illinois, 83 U.S. (16 Wall.) 130 (1873).

Dothard v. Rawlinson, 433 U.S. 321 (1977).

Emel Boyraz v. Turkey, Application No. 61960/08 (Eur. Ct. H.R. Dec. 2, 2014).

Johnson v. Transportation Agency, U.S. 616 (1987).

Kalanke v. Bremen, European Court 450/93 (1995).

Karlheinz Schmidt v. Germany, Application No. 13580/88 (Eur. Ct. H.R. Jul. 18, 1994).

Markin v. Russia, Application No. 30078/06 (Eur. Ct. H.R. Mar. 22, 2012).

Marschall v. Land Nordrhein-Westfalen, European Court case 409/95 (1997).

Mississippi University for Women v. Hogan, 458 U.S. 718, 735 (1982).

Personnel Administrator of Massachusetts v. Feeney, 442 U.S. 256, 273, 276 (1979).

Petrovic v. Austria, Application No. 20458/92 (Eur. Ct. H.R. Mar. 27, 1998).

Phillips v. Martin Marietta Corp., 400 U.S. 542, 543 (1971).

Price Waterhouse v. Hopkins, 490 U.S. 228 (1989).

Roca Álvarez v. Sesa Start España ETT SA, European Court case 104/09 (2010).

Rostker v. Goldberg, 453 U.S. 57 (1981).

Schlesinger v. Ballard, 419 U.S. 498, 508 (1975).

Texas Dept. of Community Affairs v. Burdine, 450 U.S. 248, 253 (1981).

UAW v. Johnson Controls, Inc., 499 U.S. 187, 211 (1991).

United States v. Burke, 504 U.S. 229, 254 (1992).

U.S. v. Virginia, 518 U.S. 515 (1996).

Vorchheimer v. School District of Philadelphia, 430 U.S. 703 (1977).

Wal-Mart v. Dukes, 564 U.S. _ (2011).

Washington v. Gunther, 452 U.S. 161 (1981).

Wimberly v. Missouri Labor and Industrial Relations Commission, 479 U.S. 511 (1987).

Young v. UPS, 575 U.S. _ (2015).
