# Supplementary material for: “Why Can’t I Become a Manager?”—A Systematic Review of Gender Stereotypes and Organizational Discrimination
Source: Int J Environ Res Public Health. 2019 May 22;16(10):1813. doi: 10.3390/ijerph16101813 (PMC6572654; doi:10.3390/ijerph16101813)
Supplement: Supplementary file 1 [file ijerph-16-01813-s001.zip › S3_References of articles included in the review of meta-analyses.docx]

**S3_References of articles included in the review of meta-analyses**

1. Badura KL, Grijalva E, Newman DA, Yan TT, Jeon G. Gender and leadership emergence: A meta-analysis and explanatory model. Pers Psychol. **2018**,71(3), 335-67. doi: 10.1111/peps.12266

2. Eagly AH, Johannesen-Schmidt MC, van Engen ML. Transformational, transactional, and laissez-faire leadership styles: A meta-analysis comparing women and men. Psychol Bull. **2003**, 129(4),569-91. doi: 10.1037/0033-2909.129.4.569

3. Eagly AH, Karau SJ, Johnson BT. Gender and Leadership Style among School Principals: A Meta-Analysis. Educ Adm Q. **1992**, 28(1), 76-102. doi: 10.1177/0013161X92028001004

4. Eagly AH, Karau SJ, Makhijani MG. Gender and the effectiveness of leaders: A meta-analysis. Psychol Bull. **1995,** 117(1), 125-45. doi: 10.1037/0033-2909.117.1.125

5. Eagly AH, Makhijani MG, Klonsky BG. Gender and the evaluation of leaders: A meta-analysis. Psychol Bull. **1992**, 111(1), 3-22. doi: 10.1037/0033-2909.111.1.3

6. Grijalva E, Newman DA, Tay L, Donnellan MB, Harms PD, Robins RW, et al. Gender differences in narcissism: A meta-analytic review. PsycholBull. **2015**, 141(2), 261-310. doi: 10.1037/a0038231

7. Hoobler JM, Masterson CR, Nkomo SM, Michel EJ. The Business Case for Women Leaders: Meta-Analysis, Research Critique, and Path Forward. Journal of Management. **2016**, 44(6), 2473-99. doi: 10.1177/0149206316628643

8. Hosoda M, Stone-Romero EF, Coats G. The effects of physical attractiveness on job-related outcomes: a meta-analysis of experimental studies. Pers Psychol. **2003**, 56(2), 431-62. doi: 10.1111/j.1744-6570.2003.tb00157.x

9. Hyde JS. The gender similarities hypothesis. Am Psychol. **2005**, 60(6), 581-92. doi: 10.1037/0003-066X.60.6.581

10. Jeong S-H, Harrison DA. Glass Breaking, Strategy Making, and Value Creating: Meta-Analytic Outcomes of Women as CEOs and TMT members. Acad Manage J. **2016**, 60(4), 1219-52. doi: 10.5465/amj.2014.0716

11. Jones KP, Sabat IE, King EB, Ahmad A, McCausland TC, Chen T. Isms and schisms: A meta-analysis of the prejudice-discrimination relationship across racism, sexism, and ageism. J Organ Behav. **2017**, 38(7), 1076-110. doi: 10.1002/job.2187

12. Koch AJ, D’Mello SD, Sackett PR. A meta-analysis of gender stereotypes and bias in experimental simulations of employment decision making. J Appl Psychol. **2015**, 100(1), 128-61. doi: 10.1037/a0036734

13. Koenig AM, Eagly AH, Mitchell AA, Ristikari T. Are leader stereotypes masculine? A meta-analysis of three research paradigms. Psychol Bull. **2011**, 137(4), 616-42. doi: 10.1037/a0023557

14. Kugler KG, Reif JAM, Kaschner T, Brodbeck FC. Gender differences in the initiation of negotiations: A meta-analysis. Psychol Bull. **2018**, 144(2), 198-222. doi: 10.1037/bul0000135

15. Nguyen H-HD, Ryan AM. Does stereotype threat affect test performance of minorities and women? A meta-analysis of experimental evidence. J Appl Psychol. **2008**, 93(6), 1314-34. doi: 10.1037/a0012702

16. Paustian-Underdahl SC, Walker LS, Woehr DJ. Gender and perceptions of leadership effectiveness: A meta-analysis of contextual moderators. J Appl Psychol. **2014**, 99(6), 1129-45. doi: 10.1037/a0036751

17. Schneid M, Isidor R, Li C, Kabst R. The influence of cultural context on the relationship between gender diversity and team performance: a meta-analysis. Int J Hum Resource Manag. **2015**, 26(6), 733-56. doi: 10.1080/09585192.2014.957712

18. Van Engen ML, Willemsen TM. Sex and Leadership Styles: A Meta-Analysis of Research Published in the 1990s. Psychol Rep. **2004**, 94(1), 3-18. doi: 10.2466/pr0.94.1.3-18

19. Williams MJ, Tiedens LZ. The subtle suspension of backlash: A meta-analysis of penalties for women’s implicit and explicit dominance behavior. Psychol Bull. **2016**, 142(2), 165-97. doi: 10.1037/bul0000039
