# Supplementary material for: “Why Can’t I Become a Manager?”—A Systematic Review of Gender Stereotypes and Organizational Discrimination
Source: Int J Environ Res Public Health. 2019 May 22;16(10):1813. doi: 10.3390/ijerph16101813 (PMC6572654; doi:10.3390/ijerph16101813)
Supplement: Supplementary file 1 [file ijerph-16-01813-s001.zip › S4_References of articles included in the content analysis.docx]

**S4_References of articles included in the content analysis**

1. Ayman R, Korabik K, Morris S. Is Transformational Leadership Always Perceived as Effective? Male Subordinates’ Devaluation of Female Transformational Leaders. J Appl Soc Psychol. **2009**, 39(4), 852-79. doi: 10.1111/j.1559-1816.2009.00463.x

2. Belliveau MA. Engendering Inequity? How Social Accounts Create vs. Merely Explain Unfavorable Pay Outcomes for Women. Organ Sci. **2011**, 23(4), 1154-74. doi: 10.1287/orsc.1110.0691

3. Benharda I, Brett JM, Lempereur A. Gender and Role in Conflict Management: Female and Male Managers as Third Parties. Negotiation and Conflict Management Research. **2013**, 6(2), 79-93. doi: 10.1111/ncmr.12004

4. Berkery E, Morley M, Tiernan S. Beyond gender role stereotypes and requisite managerial characteristics: From communal to androgynous, the changing views of women. Gender in Mgmt: Int J. **2013**, 28(5), 278-98. doi: 10.1108/GM-12-2012-0098

5. Berkery E, Tiernan S, Morley M. The relationship between gender role stereotypes and requisite managerial characteristics: the case of nursing and midwifery professionals. J Nurs Manag. **2014**, 22(6), 707-19. doi: 10.1111/j.1365-2834.2012.01459.x

6. Booysen LAE, Nkomo SM. Gender role stereotypes and requisite management characteristics: The case of South Africa. Gender in Mgmt: Int J. **2010**, 25(4), 285-300. doi: 10.1108/17542411011048164

7. Boulouta I. Hidden Connections: The Link Between Board Gender Diversity and Corporate Social Performance. J Bus Ethics. **2013**, 113(2), 185-97. doi: 10.1007/s10551-012-1293-7

8. Braidford P, Stone I, Tesfaye B. Gender, disadvantage and enterprise support – lessons from women’s business centres in North America and Europe. Jrnl of Small Bus Ente Dev. **2013**, 20(1), 143-64. doi: 10.1108/14626001311298457

9. Broadbridge A. 25 years of retailing; 25 years of change? Reflecting on the position of women managers. Gender in Mgmt: Int J. **2010**, 25(8), 649-60. doi: 10.1108/17542411011092318

10. Brody CJ, Rubin BA, Maume DJ. Gender Structure and the Effects of Management Citizenship Behavior. Soc Forces. **2014**, 92(4), 1373-404. doi: 10.1093/sf/sou017

11. Burke RJ, El‐Kot G. Gender similarities in work and well‐being outcomes among managers and professionals in Egypt. Int Jrnl of Gen and Ent. **2011**, 3(1), 56-74. doi: 10.1108/17566261111114980

12. Cabrera SF, Sauer SJ, Thomas-Hunt MC. The Evolving Manager Stereotype: The Effects of Industry Gender Typing on Performance Expectations for Leaders and Their Teams. Psychol Women Q. **2009**, 33(4), 419-28. doi: 10.1111/j.1471-6402.2009.01519.x

13. Cameron S, M. Nadler J. Gender roles and organizational citizenship behaviors: effects on managerial evaluations. Gender in Mgmt: Int J. **2013**, 28(7), 380-99. doi: 10.1108/GM-10-2012-0074

14. Carlson JH, Crawford M. Communication Stereotypes and Perceptions of Managers. SAGE Open. **2012**, 2(4), 1-10. doi: 10.1177/2158244012464978

15. Cesaroni FM, Sentuti A. Women and family businesses. When women are left only minor roles. Hist Fam. **2014**, 19(3), 358-79. doi: 10.1080/1081602X.2014.929019

16. Cook A, Glass C. Above the glass ceiling: When are women and racial/ethnic minorities promoted to CEO? Strategic Management Journal. **2014**, 35(7), 1080-9. doi: 10.1002/smj.2161

17. Elsaid AM, Elsaid E. Sex stereotyping managerial positions: A cross‐cultural comparison between Egypt and the USA. Gender in Mgmt: Int J. **2012**, 27(2), 81-99. doi: 10.1108/17542411211214149

18. Elsesser KM, Lever J. Does gender bias against female leaders persist? Quantitative and qualitative data from a large-scale survey. Hum Relat. **2011**, 64(12), 1555-78. doi: 10.1177/0018726711424323

19. García-Ael C, Cuadrado I, Molero F. Think-manager—Think-male vs. Social Role Theory: How do we perceive men and women in the labour market? Estud Psicol. **2012**, 33(3), 347-57. doi: 10.1174/021093912803758183

20. García-Ael C, Cuadrado I, Molero F. Think Manager – Think Male in Adolescents and its Relation to Sexism and Emotions in Leadership. Span J Psychol. **2013**, 16, 1-11. doi: 10.1017/sjp.2013.88

21. Gartzia L, Ryan MK, Balluerka N, Aritzeta A. Think crisis–think female: Further evidence. Eur J Work Organ Psychol. **2012**, 21(4), 603-28. doi: 10.1080/1359432X.2011.591572

22. Godoy L, Mladinic A. Estereotipos y Roles de Género en la Evaluación Laboral y Personal de Hombres y Mujeres en Cargos de Dirección. Psykhe. **2009**, 18, 51-64. doi: 10.4067/S0718-22282009000200004

23. Gregory A, Jeanes E, Tharyan R, Tonks I. Does the Stock Market Gender Stereotype Corporate Boards? Evidence from the Market’s Reaction to Directors’ Trades. Br J Manag. **2013**, 24(2), 174-90. doi: 10.1111/j.1467-8551.2011.00795.x

24. Hutchinson J, Walker E, McKenzie FH. Leadership in Local Government: ‘No Girls Allowed.’ Aust J Publ Admin. **2014**, 73(2), 181-91. doi: 10.1111/1467-8500.12075

25. Irni S. Cranky Old Women? Irritation, Resistance and Gendering Practices in Work Organizations. Gend Work Organ. **2009**, 16(6), 667-83. doi: 10.1111/j.1468-0432.2009.00455.x

26. Javalgi RRG, Scherer R, Sánchez C, Pradenas Rojas L, Parada Daza V, Hwang C, et al. A comparative analysis of the attitudes toward women managers in China, Chile, and the USA. Int Journal of Emerging Mkts. **2011**, 6(3), 233-53. doi: 10.1108/17468801111144067

27. Kark R, Waismel-Manor R, Shamir B. Does valuing androgyny and femininity lead to a female advantage? The relationship between gender-role, transformational leadership and identification. Leadersh Q. **2012**, 23(3), 620-40. doi: 10.1016/j.leaqua.2011.12.012

28. Katila S, Eriksson P. He is a Firm, Strong-Minded and Empowering Leader, but is She? Gendered Positioning of Female and Male CEOs. Gend Work Organ. **2013**, 20(1), 71-84. doi: 10.1111/j.1468-0432.2011.00570.x

29. King EB, Botsford W, Hebl MR, Kazama S, Dawson JF, Perkins A. Benevolent Sexism at Work: Gender Differences in the Distribution of Challenging Developmental Experiences. J Manag. **2010**, 38(6), 1835-66. doi: 10.1177/0149206310365902

30. Koca C, Arslan B, Aşçı FH. Attitudes towards Women’s Work Roles and Women Managers in a Sports Organization: The Case of Turkey. Gend Work Organ. **2011**, 18(6), 592-612. doi: 10.1111/j.1468-0432.2009.00490.x

31. Koenig AM, Eagly AH, Mitchell AA, Ristikari T. Are leader stereotypes masculine? A meta-analysis of three research paradigms. Psychol Bull. **2011**, 137(4), 616-42. doi: 10.1037/a0023557

32. Kusterer HL, Lindholm T, Montgomery H. Gender typing in stereotypes and evaluations of actual managers. Journal of Managerial Psych. **2013**, 28(5), 561-79. doi: 10.1108/JMP-01-2013-0012

33. Latu IM, Stewart TL, Myers AC, Lisco CG, Estes SB, Donahue DK. What We “Say” and What We “Think” About Female Managers: Explicit Versus Implicit Associations of Women With Success. Psychol Women Q. **2011**, 35(2), 252-66. doi: 10.1177/0361684310383811

34. Leskinen EA, Cortina LM. Dimensions of Disrespect: Mapping and Measuring Gender Harassment in Organizations. Psychol Women Q. **2013**, 38(1), 107-23. doi: 10.1177/0361684313496549

35. Lyness KS, Judiesch MK. Gender Egalitarianism and Work–Life Balance for Managers: Multisource Perspectives in 36 Countries. Appl Psychol. **2014**, 63(1), 96-129. doi: 10.1111/apps.12011

36. Madera JM, Hebl MR, Martin RC. Gender and letters of recommendation for academia: Agentic and communal differences. J Appl Psychol. **2009**, 94(6), 1591-9. doi: 10.1037/a0016539

37. Marco R. Gender and economic performance: Evidence from the Spanish hotel industry. Int J Hosp Manag. **2012**, 31(3), 981-9. doi: 10.1016/j.ijhm.2011.12.002

38. Maxfield S, Shapiro M, Gupta V, Hass S. Gender and risk: women, risk taking and risk aversion. Gender in Mgmt: Int J. **2010**, 25(7), 586–604. doi: 10.1108/17542411011081383

39. Melero E. Are workplaces with many women in management run differently? J Bus Res. **2011**, 64(4), 385–93. doi: 10.1016/j.jbusres.2010.01.009

40. Nemoto K. Long Working Hours and the Corporate Gender Divide in Japan. Gend Work Organ. **2013**, 20(5), 512-27. doi: 10.1111/j.1468-0432.2012.00599.x

41. Newman CJ, de Vries DH, d’Arc Kanakuze J, Ngendahimana G. Workplace violence and gender discrimination in Rwanda’s health workforce: Increasing safety and gender equality. Hum Resour Health. **2011**, 9(1), 19. doi: 10.1186/1478-4491-9-19

42. Nguyen TLH. Barriers to and facilitators of female Deans’ career advancement in higher education: an exploratory study in Vietnam. High Educ. **2013**, 66(1), 123-38. doi: 10.1007/s10734-012-9594-4

43. Okafor EE, Fagbemi AO, Hassan AR. Barriers to women leadership and managerial aspirations in Lagos, Nigeria: An empirical analysis. Afr J Bus Manage. **2011**, 5(16), 6717-26. doi: 10.5897/AJBM10.1250

44. Olidi CS, Parejo SP, Padilla MAS. The Path is Drawn Between Obstacles? The professional Career of a Female Manager. Psicol Org Trab. **2013**, 13(1), 75-88.

45. Paris LD, Howell JP, Dorfman PW, Hanges PJ. Preferred leadership prototypes of male and female leaders in 27 countries. J Int Bus Stud. **2009**, 40(8), 1396-405. doi: 10.1057/jibs.2008.114

46. Paris LD, Decker DL. Sex role stereotypes: does business education make a difference? Gender in Mgmt: Int J. **2012**, 27(1), 36-50. doi: https://doi.org/10.1108/17542411211199264

47. Post C, DiTomaso N, Lowe SR, Farris GF, Cordero R. A few good women: Gender differences in evaluations of promotability in industrial research and development. Journal of Managerial Psych. **2009**, 24(4), 348-71. doi: 10.1108/02683940910952723

48. Powell GN. The gender and leadership wars. Organ Dyn. **2011**, 40(1), 1-9. doi: 10.1016/j.orgdyn.2010.10.009

49. Reinhard M-A, Schindler S, Stahlberg D, Messner M, Mucha N. “I Don’t Know Anything About Soccer.” Swiss J Psychol. **2011**, 70(3), 149-54. doi: 10.1024/1421-0185/a000050

50. Ryan MK, Haslam SA, Hersby MD, Bongiorno R. Think crisis–think female: The glass cliff and contextual variation in the think manager–think male stereotype. J Appl Psychol. **2011**, 96(3), 470-84. doi: 10.1037/a0022133

51. Schulz DJ, Enslin C. The Female Executive’s Perspective on Career Planning and Advancement in Organizations: Experiences With Cascading Gender Bias, the Double-Bind, and Unwritten Rules to Advancement. SAGE Open. **2014**, 4(4), 1-9. doi: 0.1177/2158244014558040

52. Schwarzwald J, Koslowsky M, Bernstein J. Power tactic usage by gender at work and home: past, present, and future. Int Jnl of Conflict Management. **2013**, 24(3), 307-24. doi: 10.1108/IJCMA-03-2011-0020

53. Stoker JI, Van der Velde M, Lammers J. Factors Relating to Managerial Stereotypes: The Role of Gender of the Employee and the Manager and Management Gender Ratio. J Bus Psychol. **2012**, 27(1), 31-42. doi: 10.1007/s10869-011-9210-0

54. Taylor SN, Hood JN. It may not be what you think: Gender differences in predicting emotional and social competence. Hum Relat. **2010**, 64(5), 627-52. doi: 10.1177/0018726710387950

55. Thoroughgood CN, Sawyer KB, Hunter ST. Real Men Don’t Make Mistakes: Investigating the Effects of Leader Gender, Error Type, and the Occupational Context on Leader Error Perceptions. J Bus Psychol. **2013**, 28(1), 31-48. doi: 10.1007/s10869-012-9263-8

56. Timmers TM, Willemsen TM, Tijdens KG. Gender diversity policies in universities: a multi-perspective framework of policy measures. High Educ. **2010**, 59(6), 719-35. doi: 10.1007/s10734-009-9276-z

57. Tlaiss H. Women in Healthcare: Barriers and Enablers from a Developing Country Perspective. Int J Health Policy Manag. **2013**, 1(1), 23-33. doi: 10.15171/ijhpm.2013.05

58. Tonidandel S, Braddy PW, Fleenor JW. Relative importance of managerial skills for predicting effectiveness. Journal of Managerial Psych. **2012**, 27(6), 636-55. doi: 10.1108/02683941211252464

59. Webb TL, Sheeran P, Pepper J. Gaining control over responses to implicit attitude tests: Implementation intentions engender fast responses on attitude-incongruent trials. Br J Soc Psychol. **2012**, 51(1), 13-32. doi: 10.1348/014466610X532192

60. Woodhams C, Lupton B. Analysing gender-based diversity in SMEs. Scandinavian J Manag. **2009**, 25(2), 203-13. doi: 10.1016/j.scaman.2009.02.006

61. Zavyalova EK, Kosheleva SV. Gender stereotyping and its impact on human capital development in contemporary Russia. Hum Resource Dev Int. **2010**, 13(3), 341-9. doi: 10.1080/13678868.2010.483823
