# Supplementary material for: “Why Can’t I Become a Manager?”—A Systematic Review of Gender Stereotypes and Organizational Discrimination
Source: Int J Environ Res Public Health. 2019 May 22;16(10):1813. doi: 10.3390/ijerph16101813 (PMC6572654; doi:10.3390/ijerph16101813)
Supplement: Supplementary file 1 [file ijerph-16-01813-s001.zip › S1_Protocol.doc]

**S1_Protocol**

**Gender lawsuit cases analysis:**

**Objective:** Analyzing to what extent gender stereotypes impact on legal decisions.

**Databases:** WOS, SCOPUS and PsycINFO.

**Keywords for the search:**

1- “stereotype” AND “gender” AND “Supreme court”

2- “stereotype” AND “gender” AND “European court”

**Years:** from the beginning of the database records to the date of the search: 22nd January of 2019.

**Limits that apply to the search:** only Supreme Court and European lawsuit cases were considered; results referred to lawsuit cases non dealing with gender discrimination, such as racial and gender oriented discrimination, as well as books and dissertations were not included.

**Data analysis:** all the Supreme Court and European lawsuit cases dealing with gender discrimination at the workplace and stereotypes cited in the included articles were written down and analyzed taking into account the description of the case, the court decision and the relevance of the case in terms of stereotyping. Cases non dealing with gender discrimination at the workplace and federal cases were not included.

**Language:** English, Spanish.

**Meta-analysis review:**

**Objective:** Analyzing to what extend gender stereotypes could eventually impact on women presence in managerial positions by means of published meta-analytic studies.

**Databases:** WOS, SCOPUS and PsycINFO.

**Combination of k**eywords for the search:

| “meta-analy*” | AND | “gender” | AND | “stereotype” | AND | “leader” |
| --- | --- | --- | --- | --- | --- | --- |
|  |  | “female” |  | “discrimination” |  | “manag*” |
|  |  | “women” |  |  |  |  |

**Years:** from the beginning of the database records to the date of the search: 21st January of 2019.

**Limits that apply to the search:** non-meta-analytic studies, studies dealing with gender discrimination due to age or racial reasons, as well as books and dissertations were not considered.

**Data analysis:** findings regarding differences between women and men were analyzed and written down taking into account the significance of the differences and the criteria.

**Language:** English, Spanish.

**Stereotypes content analysis:**

**Objective:** achieving a full view of the broad variety of gender stereotypes cited in the literature.

**Databases:** WOS, SCOPUS and PsycINFO.

**Keywords for the search:** “woman manager” AND “gender stereotypes”.

**Years:** for the period 2009 to 2014. Saturation criterion was considered for stopping the search.

**Limits that apply to the search:** no country limits were set, and only journal articles dealing with stereotypes which may affect women’s access to managerial positions were included.

**Data analysis:** the content of the articles (i.e., introduction, results and discussion sections) was reduced and separated by units of analysis: words and pieces of text referring to gender stereotypes which could negatively affect women.

**Language:** English, Spanish.
